# Prevalence of metabolic syndrome in bus and truck drivers in Kashan, Iran

**DOI:** 10.1186/1758-5996-3-8

**Published:** 2011-05-19

**Authors:** Hamid R Saberi, Ali R Moravveji, Esmaeil Fakharian, Masoud Motalebi kashani, Ali R Dehdashti

**Affiliations:** 1 Department of Occupational Health, Kashan University of Medical Sciences, Iran; 2 Department of Community Medicine, Kashan University of Medical Sciences, Iran; 3 Trauma Research Center, Kashan University of Medical Sciences, Iran; 4 Department of Occupational Health, Semnan University of Medical Sciences, Iran

## Abstract

**Background:**

Bus and truck drivers are apparently more involved in metabolic syndrome and its complications due to their working conditions. The related impacts are not only harmful for driver's health, but also may endanger others. The present research was carried out to determine the prevalence of metabolic syndrome among bus and truck drivers in Kashan, a city in Iran.

**Materials:**

In 2007, 429 bus and truck male drivers were enrolled to this cross sectional study to examine the metabolic syndrome using ATPIII criteria. Statistical tests including Chi-Square test, T-student test and Pearson's correlation coefficient were used to analyze the data.

**Results:**

Prevalence of metabolic syndrome in subjects was 35.9%. Hypertension and diabetes were seen in 42.9% and 7% of the drivers respectively. Body mass index (BMI) in 41% of the drivers within the range of 25-30 was considered overweight and 23% of them were found to be obese. High triglyceride (53.4%) and low HDL-C levels (48.7%) were more common than other components of metabolic syndrome. A significant positive correlation was seen between BMI, diabetes, high blood pressure and metabolic syndrome (p < 0.001); but there was no positive correlation between metabolic syndrome and smoking (p < 0.06).

**Conclusion:**

High prevalence of metabolic syndrome and other relevant risk factors for coronary heart diseases (CHD) were detected among the drivers. Based on these findings, it is recommended to consider training programs, establish pertinent health regulations, and focus on the metabolic syndrome complications in high risk group to improve and maintain their quality of life and to promote their public health.

## 1. Introduction

Metabolic syndrome or X syndrome is associated with any combination of metabolic/non-metabolic disturbances including increased level of fasting blood sugar, and triglyceride, elevated blood pressure, low HDL level, and abdominal obesity. Individuals meeting at least three of the above mentioned abnormalities are labeled as having the disease [1,2]. These patients are at increased risk of cardiovascular diseases, diabetes, dyslipidaemia, stroke, osteoarthritis, some kinds of cancers, and their subsequent morbidity and mortality. The final result of these events is impairment of quality of life and a heavy burden of expenses to the health care system [1-3]. Many Studies have shown concurrent presence of metabolic disturbances in some individuals, which is usually more harmful than each single isolated problem [4]. Twenty five per cent of the adults in U.S are affected by the metabolic syndrome [5]. The incidence of metabolic syndrome among the Asian ethnic groups is not well defined whereas Asia is probably prone to the highest prevalence of diabetes and cardiovascular diseases in near future [4]. Prevalence of this syndrome is 19% in Mongolia [1], 21% in Jordan[3], 17% in Palestine[3], 24.2% in Malaysia [4], 21.17% in Taiwan [4], 12.2% in Singapore, 12% in Japan, 14.8% in China, 28.8% in India, and 28.6% and 27.8%, respectively, in male and female Koreans [5,6]. On the base of Framingham study, metabolic syndrome accounts for about 25% of the new cases of cardiovascular diseases. In the absence of diabetes, the ten-year risk of coronary heart disease is not increased by more than 20% with the metabolic syndrome. It is between 10 and 20 percent for males, and less than 10 per cent for females [6]. Although the underlying cause of metabolic syndrome is unknown, however, insulin resistance and visceral fat accumulation have been proposed as the initial drivers. Lack of congruent diagnostic criteria has resulted in report of variable prevalence of the diseases in different studies [7-10]. It has been reported that the prevalence of metabolic syndrome in the Islamic Republic of Iran is one of the highest in worldwide. Study on Lipid and Glucose among adult population in Tehran indicated metabolic syndrome in 42% of women and 24% of men with a total age-standardized prevalence of 33.7% [11]. One study suggests that 20-45% of the mortalities in Iran are due to cardiovascular diseases [12], While the mortality rate of cardiovascular diseases have reduced in most developed countries over the past 20 years [13]. Drivers are more likely to be involved with metabolic syndrome and its related complications because of their specific working conditions. The outcome may not only be harmful for the drivers, but also can be harmful to community as they play critical role in transportation and traffic sectors. Occupational stresses, physical inactivity, prolonged working hours, and inappropriate dietary habits have all been reported as the contributors to health risk factors in drivers [14,15]. Based on these facts this study was conducted to investigate the metabolic syndrome status among the bus and truck male drivers in Kashan located in central part of Iran.

## 2. Methods

This study was cross-sectional and the subjects included were 429 male bus and truck drivers that exclusively work on between cities roads, admitted to kashan health care centers for their regular medical examination in 2007. Demographic and anthropometric data including height, weight, waist circumference, and heart risk factors (high blood pressure, diabetes, dyslipidaemia and smoking) were recorded by completing a prepared questionnaire. The metabolic syndrome was defined according to the ATP III guidelines as the presence of three or more of the following components [16]: (1) abdominal obesity (WC > 102 cm); (2) a high triglyceride level (≥ 150 mg/dL); (3) a low HDL-C cholesterol level (<40 mg/dL); (4) high blood pressure (systolic > 130 mm Hg or diastolic > 85 mm Hg); and (5) a high fasting plasma glucose concentration (≥ 110 mg/dL).

Subjects' weight and height were measured in kilograms and centimeters respectively. Waist circumference was measured by stadiometer in the upper iliac crest region. Blood pressure was measured following a five minute resting period in a sitting position on the right hand repeated two times with at least five-minute interval. Blood pressures 140-159/90-99 and 160-180/100-119 were considered as mild and moderate hypertension respectively. Blood samples were collected in the morning after 12 hours of fasting. Body mass indices (BMI) at ranges <18, 18-24, 25-30, and >30 were considered orderly as thin, normal, overweight, and obese. Fasting blood sugar ≥126 mg/dl was defined as diabetes. Full confidentiality of the data collected was ensured to all the study participants and all interviews were taken after participant's consent. Data were analyzed by SPSS#14 using statistical tests Chi-Square, and T-student, along with Pearson's correlation coefficient.

## 3. Results

All the study subjects were exclusively male (as it is common for professional drivers in Iran). The average age of participants was 36.6 ± 10.7 (21-73) years. 137 (i.e. 31.9%) out of 429 drivers were between 30 and 39 years old. A number of 12 (2.8%) participants were above 60 years old. Table 1 shows clinical and Para clinical findings of all subjects. Blood pressure was marked high in 184(44%) drivers, while mild and moderately elevated blood pressure were found respectively for a number of 90(20%) and 94 (21%)study subjects. No case of severe elevated blood pressure was found. 30 (7%) drivers experienced diabetes. BMI was in the overweight range for 176 (41%), and in the range of obesity for 99 (23.1%) studied cases.

**Table 1:** Variables frequency distribution in all drivers and individuals involved in metabolic syndrome

Variables		All Drivers (n = 429)	Drivers with metabolic syndrome (n = 154)
Systolic Blood Pressure	<130 mmHg	160 (37)	95 (61.7)
	
	≥ mmHg	269 (62.7)	59 (38.3)

Diastolic Blood	<85 mmHg	118 (27.5)	76 (49.4)
	
	≥85 mmHg	311(72.5)	78 (50.6)

Blood Pressure	<140/90 mmHg	120 (28)	109 (70.8)
	
	≥140/90 mmHg	309 (72)	45 (29.2)

Fasting Glucose	<110 mg/dl	69 (16.1)	56 (36.4)
	
	≥110 mg/dl	360	98
		(83.9)	(63./6)

Triglyceride	<150 mg/dl	229 (53.4)	138 (89.6)
	
	≥150 mg/dl	200 (46.6)	16 (10.4)

HDL	<40 mg/dl	209 (48.7)	120 (77.9)
	
	≤40 mg/dl	220 (51/3)	34 (22.1)

Waist Circumference	≤102 cm	136 (31.7)	96 (63.3)
	
	≥102 cm	293 (68.3)	58 (37.7)

**Figure 1:**
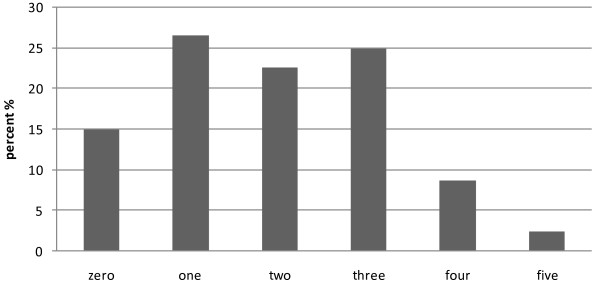
The number of ATPIII criteria in all drivers

Metabolic syndrome was met in 154 (35.9%) of individuals. The mean age of this group was 37.6(±10.5) years while in non metabolic syndrome group was 43.3(±10) years (p < 0.001). Figure 1 shows the number of ATP III component criteria in all drivers. The most common components were high level of triglyceride observed in 138 (89.6%) subjects, followed by HDL-C <40 mg/dl in 120 (77.9%) individuals. Metabolic syndrome was found in 28 (93.3%) subjects among 30 diabetic patients, as well as in 77 (64.2%) subjects among those with blood pressure above 140/90 mmHg. Furthermore 39.8% of the subjects recognized with the BMI in overweight range and 65.7% of obese persons revealed metabolic syndrome.

According to tables 2 and 3 there is a significant positive correlation between BMI, diabetes, high blood pressure and metabolic syndrome (p < 0.001). However, there was no positive correlation between metabolic syndrome and smoking (p = 0.06).

**Table 2:** Frequency distribution of metabolic syndrome by blood pressure and diabetes

Variables	Diabetes		Blood Pressure		Smoking	
**Metabolic Syndrome**	**Positive (%)**	**Negative (%)**	**high (%)**	**Normal (%)**	**Yes (%)**	**No (%)**

Positive	28 (93.3)	126 (31.6)	77 (64.2)	77 (24.9)	42 (29.8)	112 (38.9)

Negative	2 (6.7)	273 (68.4)	43 (35.8)	232 (75.1)	99 (70.2)	176 (61.1)

Total (%)	399 (100)	30 (100)	120 (100)	309 (100)	141 (100)	288 (100)

P value	<0.001		<0.001		0.065	

OR (95% CI)	30.33 (7.11-129.31)		5.39 (2.33-4.39)		0.67 (0.43-1.03)	

**Table 3:** Frequency distribution of metabolic syndrome by body mass index

Metabolic Syndrome BMI	Thin (%)	Normal (%)	Overweight (%)	Obese (%)
Positive	0 (0)	19 (13.2)	70 (39.8)	65 (65.7)

Negative	10 (100)	125 (86.8)	106 (60.2)	34 (34.3)

Total (%)	10 (100)	144 (100)	176 (100)	99 (100)

P value	<0.001			

## 3. Discussion

More than one third (35.9%) of the bus and truck male drivers participated in the study diagnosed with metabolic syndrome which reveals higher prevalence of metabolic syndrome than that of in general male population (24%) [11]. However the figure is around the prevalence in overall Iranian population (34.7%) [17]. Meanwhile the syndrome is more prevalent in women (42%). These findings imply that male drivers are more predispose to suffer from metabolic syndrome which may be related to inappropriate dietary habits and poor physical activity. Although there was not sufficient data to make a comparison of the syndrome among various job categories, our findings suggest that professional driving is a risk factor to the metabolic syndrome. In contrast some studies have already determined lower prevalence rate of metabolic syndrome in other job categories, for instance Siedlecka showed that about 11.8% of the laborers, 9.3% of the office employees, and 7.7% of the managers are likely to have metabolic syndrome [18] and in Spain this rate has reported 10.2% of the labor force [19]. These findings may to some extent be attributed to the correlation between metabolic syndrome and individuals' social duties. However, the differences in prevalence rate may also be explained by the various definitions applied to characterize the syndrome.

Our finding that indicates 42.9% of the drivers with high blood pressure is consistent with that of Targhi on 122 truck drivers in Mazandaran province, reported 36.9% of the drivers with high blood pressure [20]. The Prevalence of high blood pressure among drivers was also reported by Whiten [21], korlitez [22] and Marcinkiewicz [23] that amounts to 33%, 35% and 36.7%, respectively. In Iran figures showed that more than 15% of the adults are recognized to have high blood pressure [20]. Moreover, Isfahan Cardiovascular Research Centre has reported high blood pressure in 19% of the individuals above 19 years old [24]. The higher incidence of elevated blood pressure in the drivers may be related to their sedentary life style, nutrition and stress. A comparison between urban bus drivers and professional workers in Taiwan in 1998-1999 demonstrated high blood pressure in 56% of the drivers in contrast to 30.6% of the professional workers [25]. The difference with our study may be attributable to the differences in the study population, hypertension diagnostic criteria, environmental impacts, and dietary habits.

According to this study, the prevalence of diabetes (type II) was 7% in drivers which is higher than the rates of diabetes in Iranian population (5.5%) [26]. In our study, drivers with diabetes are significantly higher than that of other studies (2.4%) 23 and (3.5%) [27]. Different results may be explained by differences in age groups, dietary habits, gender, daily work hours or others factors.

High triglyceride level was found in 229 (53.4%) measured at the levels more than 150 mg/dl. 209 (48.7%) cases considered to have HDL-C lower than 40 mg/dl. In a survey comparing bus drivers and professional workers in Taiwan, the prevalence of high cholesterol and triglyceride levels in the drivers and professional workers was reported 34.4%, 69.4%, and 29.9%, 30.6%, respectively[25] which are similar to the results obtained from this study. The prevalence of low HDL levels is 73% and high serum triglycerides 40.6% in Iranian population [28]. In the present study increased total cholesterol (>200 mg/dl) was seen in 35.4% and high triglyceride along with low HDL in 9.9% of the population. The variation may be explained by differences in age groups, gender groups, dietary habits, and established diagnostic criteria.

This study showed that 41% and 23.1% of drivers were in the overweight and obese category, respectively. In a study conducted by Metabolism and Endocrine Glands Institution at medical university of Shahid Beheshti in Iran, 23.7% and 35% of general population in Tehran were reported to be obese and overweight, respectively [29]. Shakhatreh et al. in their study carried out in Saudi Arabia, concluded that 73.2% of the drivers were obese [30]. In Taiwan, obesity was seen in 9.6% of 2297 bus drivers while 4.6% for the workers [25]. In Poland, obesity in drivers was 17.4% [23]. In another study conducted in Mexico on professional drivers, the prevalence of obesity and overweight was 22.5 and 52.7%, respectively, which was higher than the general population of Mexico [31]. Differences in population characteristics and dietary habits may be considered as the main sources of the variation. The overuse of specific high carbohydrate, fatty foods at restaurants, sedentary life style, and lack of awareness concerning the consequences were determined responsible for the higher prevalence of obesity and overweight among drivers.

## 4. Conclusion

Metabolic syndrome is more common in drivers compared to overall population. Although, we did not evaluate dietary intake and physical activity etc but this high prevalence may be explained as a result of sedentary inactive life, high-calorie intake, high-fat dietary habits, occupational stresses, night-shift working, and lack of proper attention to health care follow up. This mandates planning for strategies to change the behavioral and dietary habits of the drivers to overcome the problem. There would be various work-related factors other than those examined in this study such as smoking habits and psychosocial factors that may affect metabolic syndrome and thus further study is required to study them. Meanwhile intervention studies should be taken to evaluate the impact of work load and dietary habits on the syndrome among drivers.

## Competing interests

The authors declare that they have no competing interests.

## Authors' contributions

HRS, ARM participated in the epidemiological and clinical work, performed statistical analysis, and together with MMK, EF drafted the manuscript. MMK, EF, DAR participated in the English language editing. All authors read and approved the final manuscript.
